# Efficacy and Safety of Foslevodopa/Foscarbidopa Monotherapy in Patients with Parkinson's Disease

**DOI:** 10.1002/mdc3.70245

**Published:** 2025-07-30

**Authors:** Jason Aldred, Manon Bouchard, Juan Carlos Martínez‐Castrillo, Michael J. Soileau, Amy M. Spiegel, Lars Bergmann, Resmi Gupta, Megha B. Shah, Pavnit Kukreja, David G. Standaert, Stuart H. Isaacson, Tove Henriksen

**Affiliations:** ^1^ Selkirk Neurology and Inland Northwest Research Spokane Washington USA; ^2^ Clinique Neuro‐Lévis and Centre de Recherche St‐Louis Lévis Québec Canada; ^3^ Department of Neurology Ramón y Cajal University Hospital Madrid Spain; ^4^ Texas Movement Disorder Specialists Georgetown Texas USA; ^5^ AbbVie Inc. North Chicago Illinois USA; ^6^ Center for Neurodegeneration and Experimental Therapeutics University of Alabama at Birmingham Birmingham Alabama USA; ^7^ Parkinson's Disease and Movement Disorders Center of Boca Raton Boca Raton Florida USA; ^8^ Department of Neurology, Movement Disorder Clinic University Hospital of Bispebjerg Copenhagen Denmark

**Keywords:** foslevodopa/foscarbidopa, monotherapy, Parkinson's disease, subcutaneous infusion

## Abstract

**Background:**

As Parkinson's disease (PD) progresses, managing symptoms becomes increasingly difficult. Foslevodopa/foscarbidopa (LDp/CDp), a 24‐hour/day continuous subcutaneous infusion of levodopa/carbidopa (LD/CD) prodrugs, improves motor complications. The feasibility and sustainability of LDp/CDp monotherapy warrants investigation.

**Objective:**

The aim was to report the efficacy and safety of LDp/CDp monotherapy and combination therapy.

**Methods:**

This post hoc analysis assessed patients with PD and ≥2.5 “Off” hours/day receiving LDp/CDp monotherapy or combination therapy in 3 trials: a 12‐week randomized active‐controlled trial (RCT) comparing LDp/CDp with oral immediate‐release LD/CD (NCT04380142), a 52‐week open‐label trial of LDp/CDp (NCT03781167), and its 96‐week open‐label extension study (OLE; NCT04379050). Monotherapy was defined as receiving LDp/CDp without concomitant PD medications; combination therapy was defined as receiving LDp/CDp with other PD medications.

**Results:**

In the RCT, 74 of 141 patients received LDp/CDp. The 52‐week trial enrolled 244 patients; 129 entered the OLE. Of LDp/CDp‐treated patients, 19 of 74 (25.7%) in the RCT, 49 of 244 (20.1%) in the 52‐week trial, and 46 of 129 (35.7%) in the OLE received monotherapy. In the RCT, mean (standard deviation) change from baseline to week 12 in “Off” time was −4.5 (4.4) and −3.0 (3.5) hours for monotherapy and combination therapy, respectively; +4.1 (3.7) and +3.1 (3.6) hours for “On” time without troublesome dyskinesia; and +4.0 (3.6) and +4.0 (3.9) hours for “On” time without dyskinesia. Efficacy was similar in open‐label trials. Improvements in the Movement Disorder Society‐Unified Parkinson's Disease Rating Scale Part II, 39‐item Parkinson's Disease Questionnaire, Parkinson's Disease Sleep Scale‐2 scores, and overall safety were comparable between monotherapy and combination therapy groups.

**Conclusions:**

LDp/CDp monotherapy treatment may be suitable for up to 96 weeks.

The pathological hallmark of Parkinson's disease (PD) is dopaminergic neuron degeneration in the substantia nigra, leading to dopamine deficiency in the basal ganglia.^1^, ^2^ The gold‐standard PD treatment is dopamine replacement therapy with oral levodopa (LD) and carbidopa (CD).^2^, ^3^, ^4^ However, as the disease progresses, the oral LD therapeutic window narrows, and plasma LD levels can fluctuate predictably and unpredictably with intermittent dosing. This fluctuation results in alternating periods of dyskinesia and “Off” time, corresponding to the peaks and troughs in LD plasma concentrations.^2^, ^3^, ^4^

Managing these symptoms often burdens patients with increasingly complex treatment regimens.^5^ Over half of patients with PD are treated with ≥2 different PD medications to control motor fluctuations as well as multiple add‐on therapies to manage nonmotor symptoms and other comorbidities.^4^, ^5^, ^6^ Polypharmacy can increase the risk of drug–drug interactions and medication intake errors, leading to further adverse effects and disease burden.^4^

Polypharmacy is also linked to a substantial subjective pill burden. Patients often need to take dopaminergic medications 3 or 4 times per day; patients at advanced stages may require as many as 6 to 10 doses each day.^5^ Nighttime PD medication use can prevent some patients from having uninterrupted sleep, and patients may still experience early morning “Off” time before their first morning dose of LD takes effect.^7^ Several recent studies have found that pill burden presents a significant challenge for patients with PD and suggest that a simpler treatment regimen (eg, monotherapy) may be clinically valuable.^8^, ^9^, ^10^, ^11^, ^12^, ^13^, ^14^

Greater treatment regimen complexity is associated with decreased patient adherence, leading to poorer symptom control.^15^ Results from a multicenter European study showed that patients who took less than 80% of their prescribed medication doses had significantly worse motor scores and significantly greater “Off” time than those who were adherent^16^; however, even those patients who remain adherent to complex medical regimens can develop poor motor control.^3^, ^4^ Additionally, nonadherence can negatively affect care partners who must manage increasing care requirements as the patient's condition deteriorates.^15^ Furthermore, nonadherence can increase health‐care system burden.^5^, ^17^ A simplified treatment regimen that can adequately control PD symptoms, reduce pill burden, enhance adherence, and improve patient outcomes is needed.

Foslevodopa/foscarbidopa (LDp/CDp), a soluble formulation of LD/CD prodrugs delivered as a 24‐hour/day continuous subcutaneous infusion (CSCI), quickly achieves and maintains consistent, predictable steady‐state plasma levels of LD.^2^, ^18^ In a phase 3, double‐blind, randomized, active‐controlled trial (RCT), LDp/CDp led to a statistically significant increase in “On” time without troublesome dyskinesia and a decrease in “Off” time (*P* < 0.01), demonstrating superiority over oral immediate‐release LD/CD.^18^ In a 52‐week, single‐arm, open‐label, phase 3 trial, LDp/CDp improved motor symptoms, PD‐related sleep disturbances, and quality of life (QoL) versus baseline.^19^ The motor symptom improvements observed in the 52‐week trial were sustained over its 96‐week, open‐label extension (OLE).^20^ In all 3 studies, 24‐hour/day CSCI of LDp/CDp demonstrated a favorable benefit–risk profile in patients with advanced PD.^18^, ^19^, ^20^

Although results from clinical trials have demonstrated the efficacy and safety of LDp/CDp, its potential as a monotherapy for PD requires further investigation. We present post hoc data from three phase 3 clinical trials and report the efficacy and safety of LDp/CDp administered as monotherapy or in combination with other PD medications.

## Patients and Methods

### Study Design and Patients

This post hoc analysis assessed patients with PD who received LDp/CDp monotherapy or combination therapy in three phase 3 clinical trials. These trials included a 12‐week RCT comparing LDp/CDp with oral immediate‐release LD/CD (NCT04380142); a 52‐week, single‐arm, open‐label trial of LDp/CDp (NCT03781167); and a 96‐week OLE of the 52‐week parent trial (NCT04379050).^18^, ^19^, ^20^ The 12‐week RCT was a phase 3, double‐blind, double‐dummy, active‐controlled, multicenter trial conducted across 65 academic and community sites in the United States and Australia. Patients aged ≥30 years with idiopathic, LD‐responsive PD; an average “Off” time of ≥2.5 hours per day; preserved cognitive function (Mini‐Mental State Examination score ≥24); and an LD equivalent daily dose of ≥400 mg were eligible to participate. Patients were randomly assigned 1 to 1 to receive CSCI of LDp/CDp plus oral capsules of placebo or CSCI of placebo solution plus oral encapsulated immediate‐release LD/CD tablets. The double‐blind treatment period included a 4‐week optimization phase and an 8‐week maintenance phase. LDp/CDp infusion rate changes were allowed only during the optimization phase; concomitant PD medication changes were not permitted during the 12‐week treatment period unless investigators deemed it medically necessary.

The 52‐week, open‐label trial was a phase 3, single‐arm trial conducted at 60 sites across 13 countries. Patients aged ≥30 years with idiopathic, LD‐responsive PD; an average “Off” time of ≥2.5 hours per day; and preserved cognitive function were included. The trial included a 4‐week optimization period, in which the investigator could adjust concomitant PD medications to optimize clinical response, followed by a 48‐week maintenance period. Investigators determined treatment regimens, and no directive was issued by the sponsor to pursue LDp/CDp monotherapy. The investigator could adjust the hourly LDp/CDp infusion rate at any point during the study. Patients who completed the 52‐week treatment period were eligible to enroll in the 96‐week OLE. The OLE remains ongoing; data analyzed here were from an interim cutoff date of August 17, 2022. Medications containing LD/CD (except for rescue) and catechol‐*O*‐methyltransferase inhibitors were not permitted during the studies; LDp/CDp replaced these medications in patients' treatment regimens. In the 12‐week study, the extra dose functionality of the pump was disabled to prevent unmasking, and rescue was enabled with oral medication; the 52‐ and 96‐week studies allowed extra doses to be administered by the pump.

Independent ethics committees or institutional review boards at each study site approved study protocols, informed consent forms, and recruitment materials before patient enrollment. All studies were conducted in accordance with the International Council for Harmonisation guidelines, applicable regulations, and the Declaration of Helsinki. All patients were informed of the study details and provided written informed consent.

### Assessments and Analysis

Efficacy endpoints included mean change from baseline to final available visit in “Off” time, “On” time without troublesome dyskinesia (defined as “On” time without dyskinesia plus “On” time with nontroublesome dyskinesia), and “On” time without dyskinesia, based on entries in the PD diary. A valid PD diary day was defined as a day with ≤2 hours of missing data (ie, ≤4 missing entries) or ≥12 awake hours (ie, at least 24 “Off” or “On” time entries) for the entire 24‐hour diary.

Additional efficacy endpoints included mean change from baseline to final available visit in motor aspects of experiences of daily living as assessed using the Movement Disorder Society‐Unified Parkinson's Disease Rating Scale (MDS‐UPDRS) Part II score, PD‐related sleep symptoms as measured using the Parkinson's Disease Sleep Scale‐2 (PDSS‐2) total score, and QoL as assessed using the 39‐item Parkinson's Disease Questionnaire (PDQ‐39) summary index. For efficacy analyses, the baseline of the 96‐week OLE corresponded to the start of the 52‐week trial.

Safety was analyzed for all patients who received ≥1 dose of study drug. Adverse events were coded using the *Medical Dictionary for Regulatory Activities* (*MedDRA*), version 24.0, in the 12‐week RCT and the 52‐week trial; and *MedDRA*, version 25.1, in the 96‐week OLE.

Efficacy and safety were analyzed by the type of therapy received. LDp/CDp monotherapy was defined as receiving no concomitant PD medications. Use of oral LD/CD as rescue therapy during the 12‐week trial and as rescue therapy and/or loading dose in the 52‐week trial and the 96‐week OLE was permitted and did not change monotherapy status. This loading dose was given at LDp/CDp treatment initiation or if dosing was discontinued for ≥3 hours. Combination therapy was defined as treatment with LDp/CDp in combination with any PD medication (eg, monoamine oxidase‐B inhibitors, dopamine agonists, anticholinergics, amantadine). Oral LD/CD monotherapy was defined as not receiving any concomitant PD medications throughout the entire trial. The LDp/CDp regimen used in the 52‐week trial could be maintained (as monotherapy or combination therapy) or modified (from monotherapy to combination therapy or vice versa) at the investigator's discretion at baseline of the 96‐week OLE.

### Statistics

Efficacy measures were summarized by reporting the mean and standard deviation (SD) of the change from baseline to the final available visit. For the 12‐week RCT, a repeated‐measures model was used to assess the change from baseline to week 12, and *P*‐values were used to indicate a significant difference from baseline. For the 52‐week trial and 96‐week OLE, *P‐*values were obtained using a 2‐sided, paired‐sample *t*‐test and were used to indicate significant differences between the final available visit and the baseline visit. Statistical comparisons were not made between monotherapy and combination therapy groups within each study as these analyses may not be robust or generalizable due to limited patient numbers in some groups. Safety assessments were summarized by reporting the number and percentage of patients with treatment‐emergent adverse events (TEAE) among all patients who received any study drug. TEAEs were reported by the *MedDRA* system organ class and preferred term. For all models, statistical significance was set at the 0.05 level.

## Results

### Patients

Of the 141 patients in the 12‐week RCT, 74 were randomly assigned to LDp/CDp and 67 to oral immediate‐release LD/CD. In the 52‐week trial, 244 patients were enrolled, 137 completed the study, and 129 entered the OLE.

Baseline demographics and characteristics were generally balanced between groups for LDp/CDp monotherapy and combination therapy in the 3 clinical trials (Table 1). The mean (SD) age ranged from 64.3 (10.7) to 67.4 (9.6) years among patients who received LDp/CDp monotherapy across the 3 trials and from 62.6 (8.3) to 65.9 (9.1) years for those who received LDp/CDp combination therapy. Among patients in the LDp/CDp monotherapy groups, 54.3% to 59.2% were men; in the combination therapy groups, 60.0% to 70.9% were men. Across all trials, more than 80% of patients in both groups were White. The proportion of patients who had PD for ≥10 years ranged from 40.8% to 54.3% in the LDp/CDp monotherapy groups and from 47.3% to 64.6% in the combination therapy groups. The mean (SD) time since the onset of motor fluctuations was 4.8 (2.9) to 6.0 (4.6) years in the LDp/CDp monotherapy groups and 5.9 (4.1) to 6.8 (4.8) years in the combination therapy groups.

**TABLE 1:** Baseline demographic and clinical characteristics

	12‐week RCT				52‐week trial		96‐week OLE*	
	Oral LD/CD (n = 67)		LDp/CDp (n = 74)		LDp/CDp (N = 244)		LDp/CDp (N = 127)**	
Characteristic	Mono (n = 22)	Combo (n = 45)	Mono (n = 19)	Combo (n = 55)	Mono (n = 49)	Combo (n = 195)	Mono (n = 46)	Combo (n = 81)**
Age (years)	67.0 (10.4)	66.5 (9.7)	67.4 (9.6)	65.9 (9.1)	64.8 (9.4)	63.7 (9.1)	64.3 (10.7)	62.6 (8.3)
Sex, n (%)								
Female	5 (22.7)	13 (28.9)	8 (42.1)	16 (29.1)	20 (40.8)	78 (40.0)	21 (45.7)	25 (30.9)
Male	17 (77.3)	32 (71.1)	11 (57.9)	39 (70.9)	29 (59.2)	117 (60.0)	25 (54.3)	56 (69.1)
Race, n (%)								
White	19 (86.4)	42 (93.3)	17 (89.5)	53 (96.4)	44 (89.8)	163 (83.6)	42 (91.3)	68 (84.0)
Black	1 (4.5)	1 (2.2)	1 (5.3)	1 (1.8)	1 (2.0)	0	0	0
Asian	2 (9.1)	1 (2.2)	0	0	3 (6.1)	31 (15.9)	4 (8.7)	12 (14.8)
Other^a^	0	1 (2.2)	1 (5.3)	1 (1.8)	1 (2.0)	1 (0.5)	0	1 (1.2)
PD duration, n (%)								
<10 years	12 (54.5)	20 (44.4)	11 (57.9)	29 (52.7)	29 (59.2)	69 (35.4)	21 (45.7)	32 (39.5)
≥10 years	10 (45.5)	25 (55.6)	8 (42.1)	26 (47.3)	20 (40.8)	126 (64.6)	25 (54.3)	49 (60.5)
Time since onset of motor fluctuation (years)	5.0 (3.7)	5.8 (4.3)	4.8 (2.9)	5.9 (4.1)	5.9 (4.3)	6.8 (4.8)^b^	6.0 (4.6)	6.6 (4.3)^c^
Absolute “Off” time (hours)	6.4 (2.4)	5.9 (1.9)	6.8 (2.7)	6.4 (1.9)^d^	6.4 (2.0)^e^	6.0 (2.5)^f^	2.6 (2.8)^g^	2.5 (2.9)^h^
Absolute “On” time without troublesome dyskinesia (hours)	8.5 (3.5)	10.3 (2.4)	8.9 (3.8)	9.9 (2.4)^d^	8.4 (2.9)^e^	9.6 (2.6)^f^	12.8 (3.5)^g^	13.6 (3.6)^h^
Absolute “On” time without dyskinesia (hours)	7.1 (4.3)	7.8 (3.5)	7.2 (4.1)	7.7 (3.3)^d^	6.4 (3.3)^e^	6.7 (3.5)^f^	11.1 (4.3)^g^	11.0 (5.5)^h^
MDS‐UPDRS Part II score	14.7 (7.4)	12.6 (5.8)	15.9 (7.1)	15.1 (6.9)	16.3 (7.4)	15.6 (7.4)	13.2 (7.0)^i^	11.3 (7.0)^j^
PDQ‐39 summary index total score	31.3 (17.1)	23.6 (12.4)^k^	33.8 (18.8)	29.6 (15.0)^d^	37.4 (14.6)	33.8 (15.0)^b^	27.5 (16.0)^i^	25.2 (15.7)^j^
PDSS total score	19.2 (10.6)	18.7 (8.6)^k^	22.7 (10.6)	20.7 (8.1)^l^	21.4 (9.2)	20.2 (9.8)^b^	N/A	N/A

*Note*: Data are shown as mean (standard deviation) unless indicated otherwise.

Abbreviations: RCT, randomized active‐controlled trial; OLE, open‐label extension; LD/CD, levodopa/carbidopa; LDp/CDp, foslevodopa/foscarbidopa; mono, monotherapy; combo, combination therapy; MDS‐UPDRS, Movement Disorder Society‐Unified Parkinson's Disease Rating Scale; PDQ, Parkinson's Disease Questionnaire; PDSS, Parkinson's Disease Sleep Scale; N/A, not available.

* Baseline of the 96‐week trial corresponds to the end of the 52‐week trial.

** There are 2 patients for whom baseline is “missing.”

^a^ Includes American Indian or Alaska native, native Hawaiian or other Pacific Islander, or those reporting multiple races.

^b^ n = 194.

^c^ n = 80.

^d^ n = 54.

^e^ n = 47.

^f^ n = 189.

^g^ n = 40.

^h^ n = 69.

^i^ n = 45.

^j^ n = 76.

^k^ n = 44.

^l^ n = 53.

In the 12‐week RCT, LDp/CDp replaced a mean (SD) of 12.0 (5.7) LD pills/day, and 36.5% (27/74) of patients reduced their regimens by >12 pills/day (Fig. S1). In the 52‐week trial, LDp/CDp replaced 13.1 (5.8) LD pills/day, and 50.0% (122/244) of patients reduced their treatment by >12 pills/day.

### LDp/CDp Monotherapy

**FIG. 1:**
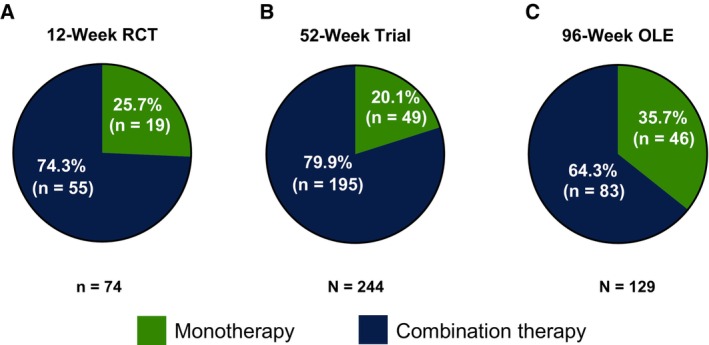
LDp/CDp monotherapy sustained throughout the (**A**) 12‐week RCT, (**B**) 52‐week trial, and (**C**) 96‐week OLE. LDp/CDp, foslevodopa/foscarbidopa; OLE, open‐label extension; RCT, randomized active‐controlled trial.

In the 12‐week RCT, 25.7% (19/74) of patients sustained treatment with LDp/CDp as monotherapy throughout the treatment period (Fig. 1A). In the 52‐week trial, LDp/CDp was sustained throughout the treatment period as monotherapy in 20.1% (49/244) of the patients (Fig. 1B), and in the 96‐week OLE, the proportion of patients receiving sustained monotherapy was 35.7% (46/129; Fig. 1C). A few patients treated with LDp/CDp monotherapy used oral LD/CD rescue doses, and at the end of the study treatment periods, daily LD rescue medication doses were <250 mg (Table S1). Among patients who continued treatment with concomitant PD medications in the OLE, approximately 40% simplified their treatment regimen by reducing the number of medication classes by ≥1, which was maintained through week 72 (Fig. S2). Patients treated with LDp/CDp who changed their dose of concomitant medications from baseline to the end of the 12‐week RCT and 52‐week trial were able to reduce the doses by an average of 0.8 to 35.6 mg of LD equivalent, though doses increased slightly (2.4 mg) in the 96‐week OLE (Table S2).

### Efficacy

**FIG. 2:**
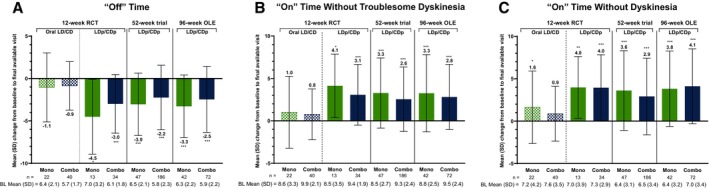
Change from baseline to final visit in (**A**) “Off” time, (**B**) “On” time without troublesome dyskinesia, and (**C**) “On” time without dyskinesia. Reported values are mean (SD [standard deviation]) change from baseline to week 12 for the RCT and mean (SD) change from baseline to final available visit for the 52‐week trial and 96‐week OLE. The baseline of the 96‐week OLE corresponds to the start of the 52‐week trial. *P ≤ 0.05, **P ≤ 0.01, and ***P ≤ 0.001 versus baseline. BL, baseline; Combo, combination therapy; LD/CD, levodopa/carbidopa; LDp/CDp, foslevodopa/foscarbidopa; Mono, monotherapy; OLE, open‐label extension; RCT, randomized active‐controlled trial.

In the 12‐week RCT, significant improvements from baseline in motor symptoms were observed with LDp/CDp when administered as monotherapy or in combination with other PD medications. The mean (SD) changes from baseline to week 12 for the monotherapy and combination therapy groups were −4.5 (4.4) and −3.0 (3.5) hours, respectively, for “Off” time (both *P* < 0.05; Fig. 2A); 4.1 (3.7) and 3.1 (3.6) hours for “On” time without troublesome dyskinesia (both *P* < 0.05; Fig. 2B); and 4.0 (3.6) and 4.0 (3.9) hours for “On” time without dyskinesia (both *P* < 0.01; Fig. 2C). Improvements from baseline in “Off” and “On” time with LDp/CDp monotherapy in the 12‐week RCT were consistent with those observed with long‐term monotherapy in the 52‐week trial and the 96‐week OLE (all *P* < 0.001; Fig. 2A–C).

**FIG. 3:**
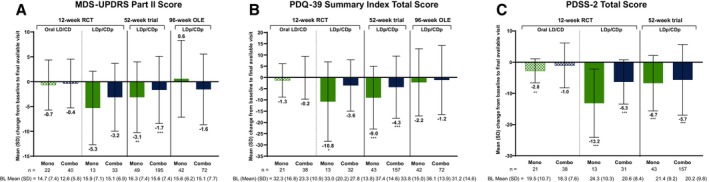
Change from baseline to final visit in (**A**) MDS‐UPDRS Part II score, (**B**) PDQ‐39 summary index total score, and (**C**) PDSS‐2 total score.^a^ Reported values are mean (SD [standard deviation]) change from baseline to final available visit except for MDS‐UPDRS Part II data for the 12‐week RCT, which is change from baseline to week 12. Baseline of the 96‐week trial corresponds to the start of the 52‐week trial. *P ≤ 0.05, **P ≤ 0.01, and ***P ≤ 0.001 versus baseline. BL, baseline; Combo, combination therapy; LD/CD, levodopa/carbidopa; LDp/CDp, foslevodopa/foscarbidopa; MDS‐UPDRS, Movement Disorder Society‐Unified Parkinson's Disease Rating Scale; Mono, monotherapy; OLE, open‐label extension; PDQ‐39, 39‐item Parkinson's Disease Questionnaire; PDSS‐2, Parkinson's Disease Sleep Scale‐2; RCT, randomized active‐controlled trial. ^a^PDSS‐2 was not evaluated in the OLE.

Improvements in motor aspects of daily living were also observed. In the 12‐week RCT, the mean (SD) change from baseline to final visit in MDS‐UPDRS Part II score was −5.3 (7.4) and −3.2 (6.8) for the LDp/CDp monotherapy and combination therapy groups, respectively (Fig. 3A). In the 52‐week trial, significant improvements from baseline to final visit in MDS‐UPDRS Part II score were observed for monotherapy and combination therapy groups (−3.1 [7.1] and −1.7 [6.8], respectively; both *P* < 0.01); small changes from baseline were observed for the 96‐week OLE (0.6 [7.7] and −1.6 [7.1], respectively) among patients who had received treatment with LDp/CDp for 52 weeks before the OLE baseline.

Similar trends were observed for improvements in QoL (Fig. 3B). Patients in the 12‐week RCT showed improvements from baseline to final visit in PDQ‐39 summary index total scores for the LDp/CDp monotherapy (mean [SD], −10.8 [17.7]; *P* < 0.05) and combination therapy (mean [SD], −3.6 [11.4]; *P‐*value not significant) groups. In the 52‐week trial, significant improvements from baseline to final visit in PDQ‐39 summary index total scores were observed for monotherapy and combination therapy groups (mean [SD], −9.0 [14.0] and −4.3 [13.8], respectively, both *P* < 0.001), whereas small improvements were observed for the 96‐week OLE among patients treated with LDp/CDp for 52 weeks before the OLE.

Sleep improvements were also observed with LDp/CDp monotherapy and combination therapy (Fig. 3C). In the 12‐week RCT, the mean (SD) change from baseline to week 12 in PDSS‐2 total score was −13.2 (11.0) and −6.3 (7.1) for the monotherapy and combination therapy groups, respectively (both *P* < 0.001). The improvements for the monotherapy and combination therapy groups were consistent with those observed in the 52‐week trial (−6.7 [8.9] and −5.7 [11.3], respectively; both *P* < 0.001). PDSS‐2 was not assessed in the 96‐week OLE.

For all assessed efficacy outcomes in the 12‐week RCT, improvements were numerically greater with LDp/CDp versus oral immediate‐release LD/CD across monotherapy and combination therapy groups.

In the 52‐week trial (Fig. S3A) and the 96‐week OLE (Fig. S3B), the mean daily LD equivalent dose was similar for the monotherapy and combination therapy patient groups (~2000 mg/day throughout the studies).

**FIG. 4:**
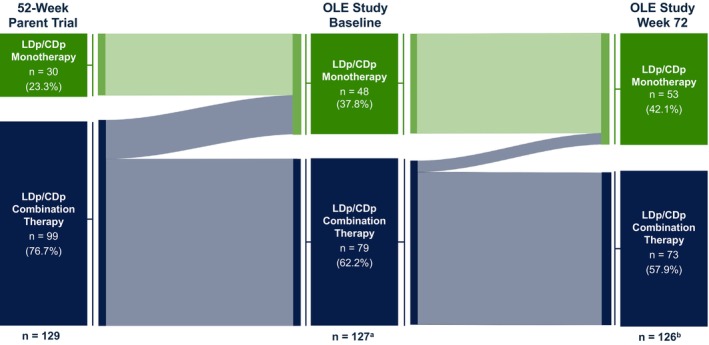
LDp/CDp treatment patterns for patients who completed the 52‐week parent trial and enrolled in the 96‐week open‐label extension. LDp/CDp, foslevodopa/foscarbidopa; OLE, open‐label extension. ^a^At baseline of the OLE study, data were missing for 2 patients receiving monotherapy. ^b^At OLE study week 72, data were missing for 2 patients receiving combination therapy and 1 patient receiving monotherapy; 1 patient at weeks 24 and 48 of the OLE study had switched from monotherapy to combination therapy, but at week 72, 0 patients with available data had switched from monotherapy to polytherapy.

Figure 4 shows LDp/CDp treatment patterns for patients who completed the 52‐week trial and enrolled in the 96‐week OLE. Of the 129 patients who completed the 52‐week parent trial and entered the 96‐week OLE, 23.3% (30/129) and 76.7% (99/129) had received LDp/CDp monotherapy and combination therapy, respectively, during the 52‐week trial. Of the 30 patients who initiated LDp/CDp as monotherapy in the 52‐week parent trial, 28 (93.3%) continued with monotherapy into the 96‐week OLE, and 2 (6.7%) had missing data at OLE baseline. Most patients receiving monotherapy (96.7% [29/30]) sustained monotherapy through OLE week 72. Almost one‐fifth (20.2% [20/99]) of patients who received LDp/CDp as combination therapy in the 52‐week trial discontinued all concomitant PD medications and switched to LDp/CDp monotherapy when entering the 96‐week OLE. By week 72 of the OLE, 4 additional patients from the combination therapy group switched to monotherapy. Most patients (73.7% [73/99]) who received LDp/CDp combination therapy continued to receive combination therapy at week 72 in the 96‐week OLE.

### Safety

Safety results were comparable between the LDp/CDp monotherapy and combination therapy groups across the trials (Table 2). In the 12‐week RCT, TEAEs were reported in 78.9% and 85.5% of patients in the LDp/CDp monotherapy and combination therapy groups, respectively. Similarly, 89.1% to 93.9% and 80.7% to 94.4% of patients in the monotherapy and combination therapy groups, respectively, experienced TEAEs in the open‐label studies. Across the 3 trials, 5.3% to 26.5% in the monotherapy groups and 10.9% to 25.6% in the combination therapy groups experienced severe TEAEs. Serious TEAEs were reported in 0% to 28.3% in the monotherapy groups and 10.9% to 26.7% in the combination therapy groups across the 3 trials. In the 12‐week RCT, 10.5% of patients in the LDp/CDp monotherapy group and 25.5% of patients in the combination therapy group experienced TEAEs that led to treatment discontinuation. In the 52‐week trial and 96‐week OLE, 8.7% to 28.6% of patients in the monotherapy groups and 6.0% to 25.6% of patients in the combination therapy groups experienced TEAEs that resulted in treatment discontinuation.

**TABLE 2:** Summary of safety results

	12‐week RCT				52‐week trial		96‐week OLE	
	Oral LD/CD (n = 67)		LDp/CDp (n = 74)		LDp/CDp (N = 244)		LDp/CDp (N = 129)	
**TEAE**,* n (%)	Mono (n = 22)	Combo (n = 45)	Mono (n = 19)	Combo (n = 55)	Mono (n = 49)	Combo (n = 195)	Mono (n = 46)	Combo (n = 83)
Any TEAE	6 (27.3)	27 (60.0)	15 (78.9)	47 (85.5)	46 (93.9)	184 (94.4)	41 (89.1)	67 (80.7)
Severe TEAE	0	1 (2.2)	1 (5.3)	6 (10.9)	13 (26.5)	50 (25.6)	11 (23.9)	18 (21.7)
Serious TEAE	0	4 (8.9)	0	6 (10.9)	11 (22.4)	52 (26.7)	13 (28.3)	22 (26.5)
TEAE leading to study drug discontinuation	0	1 (2.2)	2 (10.5)	14 (25.5)	14 (28.6)	50 (25.6)	4 (8.7)	5 (6.0)

*Note*: Data shown include all patients who received the study drug.

Abbreviations: RCT, randomized active‐controlled trial; OLE, open‐label extension; LD/CD, levodopa/carbidopa; LDp/CDp, foslevodopa/foscarbidopa; mono, monotherapy; combo, combination therapy; TEAE, treatment‐emergent adverse event.

* Patients are counted once in each row, regardless of the number of events they may have had.

The most common TEAEs were infusion site TEAEs (Table S3). Individual infusion site TEAEs and PD‐related TEAEs generally occurred at similar rates between the monotherapy and combination therapy groups for each respective study (Table S3). Hallucination rates were generally higher with LDp/CDp (0%–18.5% across trials and treatment groups) compared with oral LD/CD (0%–2.2%).

## Discussion

Findings from this post hoc analysis of three phase 3 clinical trials suggest that LDp/CDp monotherapy is achievable in a relevant proportion of patients and is associated with favorable efficacy and safety outcomes. In the 12‐week RCT, approximately one‐fourth of patients received LDp/CDp monotherapy throughout the double‐blind treatment phase, and the 52‐week trial and 96‐week OLE results showed that LDp/CDp was suitable as a long‐term monotherapy treatment for up to one‐third of patients. A higher proportion of patients sustained monotherapy in the 96‐week trial than in the other 2 trials, possibly because the shorter trials granted less flexibility for comedication changes, a potential selection bias in the cohorts, or patients in the 96‐week trial derived greater satisfaction from continuing monotherapy, perhaps due to reduced pill burden. The analysis of treatment patterns indicated that most patients with PD who initiated LDp/CDp as monotherapy can continue treatment without additional PD medications for up to 52 to 96 weeks, and patients who initially received LDp/CDp combination therapy could discontinue concomitant PD medications and switch to monotherapy. Furthermore, improvements in motor symptoms, QoL, and sleep were observed in monotherapy and combination therapy groups, consistent with results observed in the total patient populations of the RCT and 52‐week trials,^18^, ^19^ with several efficacy outcomes showing numerically stronger improvements in the monotherapy group. The long‐term improvements are less significant for MDS‐UPDRS Part II and PDQ‐39 in the 96‐week trial, possibly due to underlying disease progression becoming more of a factor than in the shorter trials. Safety results were comparable between the 2 groups across the 3 trials.

Findings from a systematic review and meta‐analysis showed that adherence rates for patients with chronic diseases were significantly lower for 2‐times‐daily, 3‐times‐daily, and 4‐times‐daily dosing regimens compared with once‐daily (oral) regimens.^21^ For patients with PD, the lower treatment adherence associated with high pill burden and polypharmacy reduces patient QoL.^4^, ^15^ Consequently, reducing pill burden and polypharmacy may be particularly important for patients as their PD progresses and leads to further cognitive and motor impairments that make adhering to complex therapeutic regimens challenging.^4^, ^5^ In our study, a substantial number of patients sustained monotherapy with LDp/CDp throughout the respective study treatment periods. Although results suggest that LDp/CDp has the potential to reduce pill burden, it may introduce a pump burden due to the need to load and refill the device, prepare and replace the infusion set, and learn and apply aseptic techniques.^22^ Patients may also have personal preferences for oral medication compared with pump therapy; however, as pump treatment overall has demonstrated superior outcomes versus oral LD/CD, allows for more stable dosing than oral medication, and may reduce the complexity of a treatment regimen (especially in patients with age‐ and disease‐related polypharmacotherapy), a pump monotherapy may be preferable. Further research on LDp/CDp use as monotherapy and its impact on pill burden and adherence is warranted.

The efficacy of long‐term monotherapy with levodopa–carbidopa intestinal gel (LCIG) for PD has also been evaluated.^23^, ^24^, ^25^ Data from the observational GLORIA and COSMOS studies showed that monotherapy with LCIG is an effective long‐term treatment option for patients with advanced PD with similar efficacy and safety/tolerability.^23^, ^24^, ^25^ In the COSMOS study, only patients who had been treated with LCIG for ≥1 year were included, ensuring selection of those who responded well and/or tolerated the treatment.^24^ Unlike previous observational studies, our analysis employed a more robust analytical approach by defining monotherapy from day 1 of treatment. Additionally, our findings are based on data collected from 3 interventional studies, enhancing the reliability of our conclusions.

A strength of this analysis is that it included data from three phase 3 clinical trials representing a large, international patient population. Including data from the 52‐week trial and its 96‐week OLE also enabled us to evaluate the effects of long‐term LDp/CDp monotherapy. A potential limitation of this analysis is the post hoc analysis and differences in each study design methodology, which reduced the interpretation of the results. Further, all studies assess changes in “On” and “Off” time based on data collected from patients' PD diaries, which may be subject to recall bias. Additional limitations include the patient population comprising mainly White patients and only those with preserved cognition, potentially limiting the generalizability of the results. Although we report the number of LD pills and concomitant classes at baseline, the clinical trials did not capture information on the total number of pills taken per day, the number of rescue pills taken per day, or whether oral LD/CD was regularly used as rescue medication. Further, data collected in the 52‐week trial and 96‐week OLE did not account for patients who dropped out of the studies over time; the results could potentially be biased toward those patients who tolerated and responded well to treatment, which may not reflect real‐world results. As these studies were conducted using a novel therapy, some early study discontinuations may have been due to suboptimal administration. Adjustments were made to the protocols for the 12‐week RCT and 52‐week studies to improve patients' experience and retention. Considerations for the initiation and maintenance of LDp/CDp to optimize outcomes based on these clinical experiences have been published.^22^ Finally, the 12‐ and the 52‐week studies granted less flexibility in terms of handling comedication than would be allowed in the real world. A real‐world observational multicountry study (ROSSINI; NCT06107426) is underway to analyze medication patterns with LDp/CDp.

Data from three phase 3 clinical trials suggest that monotherapy with LDp/CDp CSCI may be a viable therapeutic option while reducing pill burden for patients with PD. Safety results were consistent with the known safety profile of LDp/CDp.

## Author Roles

(1) Research project: A. Conception, B. Organization, C. Execution; (2) Statistical analysis: A. Design, B. Execution, C. Review and critique; (3) Manuscript preparation: A. Writing of the first draft, B. Review and critique.

J.A.: 1B, 1C, 2C, 3A, 3B

M.B.: 1B, 1C, 2C, 3B

J.C.M.‐C.: 1B, 1C, 2C, 3B

M.J.S.: 1B, 1C, 2C, 3B

A.M.S.: 1B, 1C, 2C, 3B

L.B.: 2A, 2C, 3A, 3B

R.G.: 2A, 2B, 2C, 3B

M.B.S.: 2A, 2C, 3B

P.K.: 2A, 2C, 3B

D.G.S.: 1C, 2C, 3A, 3B

S.H.I.: 1C, 2C, 3B

T.H.: 1C, 2C, 3A, 3B

## Disclosures

**Ethical Compliance Statement:** Study protocols, informed consent forms, and recruitment materials were approved by independent ethics committees or institutional review boards at each study site before patient enrollment. All studies were conducted in accordance with the International Council for Harmonisation guidelines, applicable regulations, and the Declaration of Helsinki. All patients were informed of the study details and provided written informed consent. We confirm that we have read the journal's position on issues involved in ethical publication and affirm that this work is consistent with those guidelines.

**Funding Sources and Conflicts of Interest:** AbbVie funded this study and participated in the study design, research, analysis, data collection, interpretation of data, review, and approval for publication. No honoraria or payments were made for authorship. Medical writing support was provided by Tara Rachinsky, PhD, Alicia Salinero, PhD, ISMPP CMPP, and Jay Parekh, PharmD, ISMPP CMPP, of JB Ashtin. J.A. is a study investigator for AbbVie, and he has received honorarium from AbbVie. M.B. has received research support and honoraria for consultancy, lectures, and advisory boards from AbbVie. J.C.M.‐C. has received travel grants, research grants, and honoraria as a speaker from AbbVie. He has participated in advisory boards for AbbVie. M.J.S. has received advisory/consulting fees from AbbVie and has received research support from AbbVie. He has served on the speaking bureau for AbbVie. A.M.S., L.B., R.G., M.B.S., and P.K. are full‐time employees of AbbVie, and may hold AbbVie stock and/or stock options. D.G.S. is an investigator for studies funded by AbbVie. He has served as a consultant for or received honoraria from AbbVie. S.H.I. has received honoraria from AbbVie. T.H. has served as a speaker for AbbVie and is a primary investigator for the AbbVie M15‐741 and M15‐737 studies.

**Financial Disclosures for the Previous 12 Months:** J.A. is a study investigator for AC Immune, Annovis, Aptinyx, AstraZeneca, Atara, Athira, Biogen, Biovie, Boston Scientific, Celgene, Cerevance, Cerevel, Denali, EIP, Eli Lilly, Impax, Inhibikase, IRL Therapeutics, Merz, Neuraly, Neurocrine, Neuoderm, Novartis, PD Gene/PSG, Praxis, Revance, Roche/Genentech, Sage, Sanofi/Genzyme, Scion Neurostem, Takeda, Theravance, Triplet/HSG, and UCB. He has received honorarium from Abbott, Allergan (now AbbVie), Boston Scientific, Medtronic, Teva, and US World Meds. M.B. has received honoraria for consultancy, lectures, and advisory boards from Eli Lilly and Pfizer. She has received research support from ES Therapeutics, Biohaven, and Pfizer. J.C.M.‐C. has received honoraria as a speaker from Allergan (now AbbVie), Bial, Ipsen, Italfarmaco, Krka, Lundbeck, Medtronic, Merz, Teva, UCB, and Zambon; travel grants from Allergan (now AbbVie), Bial, Italfarmaco, Krka, Merz, UCB, and Zambon; and research grants from Allergan (now AbbVie), Italfarmaco, Lundbeck, Merz, UCB, and Zambon. He has participated in advisory boards for Bial, Ipsen, Italfarmaco, Lundbeck, Merz, Orion, Stada, UCB, and Zambon. M.J.S. has received advisory/consulting fees from Abbott, Amneal, Merz, Medtronic, Neurocrine, and Supernus; and has received research support from AbbVie, Cerevel, CND Life Sciences, Intra‐Cellular, Jazz, Praxis Precision Medicine, Scion, and Teva. He has served on the speaking bureau for Amneal, Biogen, and Kyowa Kirin, and has also received grant support from the HDSA. A.M.S., L.B., R.G., M.B.S., and P.K. declare that there are no additional disclosures to report. D.G.S. is a member of the faculty of the University of Alabama at Birmingham and is supported by endowment and university funds. He is an investigator in studies funded by the American Parkinson Disease Association, the Michael J. Fox Foundation for Parkinson Research, the National Parkinson Foundation, the Alabama Department of Commerce, the Alabama Innovation Fund, Genentech, the Department of Defense, and NIH grants P50NS108675 and R25NS079188. He has a clinical practice and is compensated for his services through the University of Alabama Health Services Foundation. He serves as deputy editor for the journal *Movement Disorders* and is compensated for this role by the International Parkinson and Movement Disorders Society. In addition, since January 1, 2022, he has served as a consultant for or received honoraria from Alnylam, Appello, Biohaven, BlueRock, Coave, Curium, F. Hoffman‐La Roche, Lilly, Sanofi‐Aventis, and Theravance. He has also received book royalties from McGraw‐Hill Publishers. S.H.I. has received honoraria for continuing medical education, consultancy services, research grants, and/or promotional speaking from Acadia, Acorda, Alexza, Amneal, Amylyx, Bial, Biogen, Biovie, Britannia, Cala, Cerevel, CND Life Sciences, Enterin, Esteve, Fasikl, Global Kinetics, Jazz, Kyowa Kirin, Lilly, Lundbeck, Merz, the Michael J. Fox Foundation, Mitsubishi Tanabe, Neurocrine, Neuroderm, Novartis, Ono, Parkinson Study Group, Pharma2B, Praxis, Revance, Roche, Rune, Sage, Sanofi, Scion, Stoparkinson, Supernus, Teva, Theravance, and UCB. T.H. has served as a speaker for Britannia, Nordic Infucare, Lundbeck, Ipsen, and Convatec. She is also a board member of a data monitoring committee at a study sponsored by Lundbeck.

## Supporting information


**Table S1.** Documented use of oral rescue LD/CD (levodopa/carbidopa) at the end of the treatment period in the monotherapy groups.
**Table S2.** Use of concomitant medications at baseline and end of the treatment period.
**Table S3.** Summary of most common TEAEs (treatment‐emergent adverse events).
**Figure S1.** Reduction in oral LD/CD pill burden.
**Figure S2.** Reduction in concomitant PD medication classes in patients receiving combination therapy in the 96‐week OLE study.
**Figure S3.** Mean daily levodopa equivalent dose in the (**A**) 52‐week trial and (**B**) 96‐week OLE.

## Data Availability

AbbVie is committed to responsible data sharing regarding the clinical trials we sponsor. This includes access to anonymized individual and trial‐level data (analysis data sets), as well as other information (eg, protocols, clinical study reports, or analysis plans), as long as the trials are not part of an ongoing or planned regulatory submission. This includes requests for clinical trial data for unlicensed products and indications. These clinical trial data can be requested by any qualified researchers who engage in rigorous, independent, scientific research and will be provided following review and approval of a research proposal, Statistical Analysis Plan (SAP), and execution of a Data Sharing Agreement (DSA). Data requests can be submitted at any time after approval in the United States and Europe and after acceptance of this manuscript for publication. The data will be accessible for 12 months, with possible extensions considered. For more information on the process or to submit a request, visit the following link: https://vivli.org/ourmember/abbvie/, then select “Home”.
