# Supplementary material for: Efficacy and Safety of Foslevodopa/Foscarbidopa Monotherapy in Patients with Parkinson's Disease
Source: Mov Disord Clin Pract. 2025 Jul 30;13(1):181–90. doi: 10.1002/mdc3.70245 (PMC12839491; doi:10.1002/mdc3.70245)

Supporting Information

Efficacy and Safety of Foslevodopa/Foscarbidopa Monotherapy in Patients with Parkinson's Disease

Jason Aldred, MD,[1] Manon Bouchard, MD,[2] Juan Carlos Martínez-Castrillo, MD, PhD,[3] Michael J Soileau, MD,[4] Amy M Spiegel, PhD,[5] Lars Bergmann, MD,[5] Resmi Gupta, PhD,[5] Megha B Shah, PharmD,[5] Pavnit Kukreja, PharmD,[5] David G Standaert, MD, PhD,[6] Stuart H Isaacson, MD,[7] and Tove Henriksen, MD[8]

[1]Selkirk Neurology & Inland Northwest Research, Spokane, Washington, United States; [2]Clinique Neuro-Lévis and Centre de Recherche St-Louis, Lévis, Québec, Canada; [3]Ramón y Cajal University Hospital, Department of Neurology. Madrid, Spain; [4]Texas Movement Disorder Specialists, Georgetown, Texas, United States; [5]AbbVie Inc., North Chicago, Illinois, United States; [6]Center for Neurodegeneration and Experimental Therapeutics, University of Alabama at Birmingham, Birmingham, Alabama, United States; [7]Parkinson's Disease and Movement Disorders Center of Boca Raton, Boca Raton, Florida, United States; [8]Department of Neurology, Movement Disorder Clinic, University Hospital of Bispebjerg, Copenhagen, Denmark

Supplementary Table 1. *Documented use of oral rescue LD/CD at the end of the treatment period in the monotherapy groups*

	12-week RCT		52-week trial	96-week OLE[a]
**Daily LD equivalent dose**, mg, mean (SD)	**Oral LD/CD**	**LDp/CDp**	**LDp/CDp**	**LDp/CDp**
Ongoing at the end of the treatment period	n = 19	n = 9	N = 26	N = 23
Rescue dose	200.0 (127.7)<br>n = 4	220.0 (142.6)<br>n = 5	100.0 (0)<br>n = 10	166.7 (115.5)<br>n = 3
Loading dose[b]	N/A	N/A	150.0 (70.7)<br>n = 2	175.0 (106.1)<br>n = 2

Abbreviations: LD/CD, levodopa/carbidopa; LDp/CDp, foslevodopa/foscarbidopa; N/A, not applicable; OLE, open-label extension; RCT, randomized active-controlled trial.
[a]Data for the 96-week OLE are reported to week 72.
[b]Only includes loading doses used if LDp/CDp infusion was discontinued for ≥3 hours.

Supplementary Table 2. *Use of concomitant medications at baseline and end of the treatment period*

	12-week RCT				52-week trial		96-week OLE	
	**Oral LD/CD (n = 67)**		**LDp/CDp (n = 74)**		**LDp/CDp (N = 244)**		**LDp/CDp (N = 129)**	
**Concomitant Medications**, n (%)	Mono	Combo	Mono	Combo	Mono	Combo	Mono	Combo
**Baseline**	n = 22	n = 45	n = 19	n = 55	n = 49	n = 195	n = 46	n = 83
No concomitant PD medications	22 (100)	1 (2.2)	19 (100)	0	36 (73.5)	13 (6.7)	38 (82.6)	4 (4.8)
Any concomitant PD medications	0	44 (97.8)	0	55 (100)	13 (26.5)	182 (93.3)	8 (17.4)	79 (95.2)
Anticholinergics	0	2 (4.4)	0	1 (1.8)	0	3 (1.5)	0	1 (1.2)
Dopaminergics	0	43 (95.6)	0	54 (98.2)	13 (26.5)	181 (92.8)	8 (17.4)	79 (95.2)
MAO-B inhibitors	0	21 (46.7)	0	26 (47.3)	0	94 (48.2)	0	37 (44.6)
Levodopa/carbidopa /entacapone	0	0	0	0	0	35 (17.9)	5 (10.9)	0
Other COMT inhibitors	0	0	0	0	1 (2.0)	13 (6.7)	0	0
Amantadine	0	15 (33.3)	0	14 (25.5)	0	63 (32.3)	0	15 (18.1)
**End of Treatment Period**[a,b]	n = 21	n = 42	n = 14	n = 36	n = 30	n = 111	n = 34	n = 55
No concomitant PD medications	21 (100)	0	14 (100)	2 (5.6)	23 (76.7)	8 (7.2)	25 (73.5)	10 (18.2)
Any concomitant PD medications	0	42 (100)	0	34 (94.4)	7 (23.3)	103 (92.8)	9 (26.5)	45 (81.8)
Anticholinergics	0	3 (7.1)	0	0	0	1 (0.9)	0	0
Dopaminergics	0	40 (95.2)	0	34 (94.4)	7 (23.3)	102 (91.9)	9 (26.5)	45 (81.8)
MAO-B inhibitors	0	21 (50.0)	0	15 (41.7)	0	47 (42.3)	0	23 (41.8)
Levodopa/carbidopa /entacapone	0	0	0	0	0	16 (14.4)	6 (17.6)	0

Other COMT inhibitors	0	0	0	0	1 (3.3)	1 (0.9)	0	0
Amantadine	0	13 (31.0)	0	8 (22.2)	0	20 (18.0)	0	7 (12.7)
**No dose change**[c]	N/A	40 (88.9)	N/A	34 (61.8)	3 (6.1)	80 (41.0)	8 (17.4)	42 (50.6)
**Dose change**[c]								
n (%)	N/A	1 (2.2)	N/A	1 (1.8)	0	14 (7.2)	0	2 (2.4)
Change in mg, mean (SD)	N/A	2.0 (N/A)	N/A	-0.8 (N/A)	N/A	-35.6 (128.9)[d]	N/A	2.4 (3.0)
**Discontinued**[c]	N/A	3 (6.7)	N/A	21 (38.2)	11 (22.4)	146 (74.9)	0	39 (47.0)

Abbreviations: Combo, combination therapy; COMT, catechol-o-methyl-transferase; LD/CD, levodopa/carbidopa; LDp/CDp, foslevodopa/foscarbidopa; MAO, monoamine oxidase; Mono, monotherapy; N/A, not applicable; OLE, open-label extension; RCT, randomized active-controlled trial.

[a]Week 9−12 for the 12-week RCT, week 40−52 for the 52-week trial, week 73−84 for the 96-week OLE.

[b]Treatment for patients in the 96-week OLE remains ongoing.

[c]n is equivalent to the baseline number of patients.

[d]n = 17.

Supplementary Table 3. *Summary of most common TEAEs*

Most common TEAEs,[a] n (%)	12-Week RCT: Oral LD/CD (n = 67) Mono (n = 22)	12-Week RCT: Oral LD/CD (n = 67) Combo (n = 45)	12-Week RCT: LDp/CDp (n = 74) Mono (n = 19)	12-Week RCT: LDp/CDp (n = 74) Combo (n = 55)	52-Week trial: LDp/CDp (N = 244) Mono (n = 49)	52-Week trial: LDp/CDp (N = 244) Combo (n = 195)	96-Week OLE: LDp/CDp (N = 129) Mono (n = 46)	96-Week OLE: LDp/CDp (N = 129) Combo (n = 83)
Infusion site								
Erythema	0	1 (2.2)	7 (36.8)	13 (23.6)	27 (55.1)	100 (51.3)	4 (8.7)	17 (20.5)
Nodule	0	0	1 (5.3)	5 (9.1)	17 (34.7)	53 (27.2)	2 (4.3)	5 (6.0)
Pain	1 (4.5)	0	4 (21.1)	15 (27.3)	7 (14.3)	31 (15.9)	2 (4.3)	3 (3.6)
Edema	0	0	3 (15.8)	6 (10.9)	13 (26.5)	34 (17.4)	0	4 (4.8)
Cellulitis	0	0	4 (21.1)	10 (18.2)	10 (20.4)	46 (23.6)	8 (17.4)	12 (14.5)
Reaction	0	0	2 (10.5)	1 (1.8)	10 (20.4)	20 (10.3)	1 (2.2)	1 (1.2)
Papule	0	0	1 (5.3)	2 (3.6)	6 (12.2)	12 (6.2)	2 (4.3)	2 (2.4)
Abscess	0	0	0	1 (1.8)	4 (8.2)	23 (11.8)	2 (4.3)	2 (2.4)
Pruritus	0	0	2 (10.5)	2 (3.6)	4 (8.2)	3 (1.5)	0	0
Induration	0	0	2 (10.5)	2 (3.6)	1 (2.0)	8 (4.1)	0	3 (3.6)
Fall	0	9 (20.0)	0	6 (10.9)	7 (14.3)	34 (17.4)	8 (17.4)	15 (18.1)
Hallucination	0	1 (2.2)	0	4 (7.3)	6 (12.2)	36 (18.5)	8 (17.4)	8 (9.6)
Anxiety	0	2 (4.4)	0	2 (3.6)	0	29 (14.9)	2 (4.3)	8 (9.6)
Dizziness	0	3 (6.7)	2 (10.5)	1 (1.8)	7 (14.3)	18 (9.2)	1 (2.2)	3 (3.6)
COVID-19	0	0	0	0	0	1 (0.5)	5 (10.9)	10 (12.0)
Dyskinesia	2 (9.1)	2 (4.4)	2 (10.5)	6 (10.9)	2 (4.1)	16 (8.2)	1 (2.2)	2 (2.4)
Catheter site bruise	0	1 (2.2)	2 (10.5)	0	0	0	0	3 (3.6)
Weight decreased	0	0	0	0	4 (8.2)	20 (10.3)	1 (2.2)	3 (3.6)
Constipation	0	0	0	3 (5.5)	5 (10.2)	15 (7.7)	4 (8.7)	3 (3.6)
Nausea	0	1 (2.2)	0	3 (5.5)	5 (10.2)	14 (7.2)	2 (4.3)	2 (2.4)
Insomnia	0	1 (2.2)	0	3 (5.5)	5 (10.2)	13 (6.7)	2 (4.3)	2 (2.4)

Note: Data shown include all patients who received study drug.
Abbreviations: Combo, combination therapy; LD/CD, levodopa/carbidopa; LDp/CDp, foslevodopa/foscarbidopa; Mono, monotherapy; OLE, open-label extension; RCT, randomized active-controlled trial; TEAE, treatment-emergent adverse event.
[a]Occurring in ≥ 10% of patients in any arm.

Supplementary Figure 1. *Reduction in oral LD/CD pill burden.*

LD/CD, levodopa/carbidopa; LDp/CDp, foslevodopa/foscarbidopa; LEDD, levodopa equivalent daily dose; RCT, randomized active-controlled trial.

[a]Calculated by converting the total LEDD at baseline into 100 mg oral LD/CD pills.

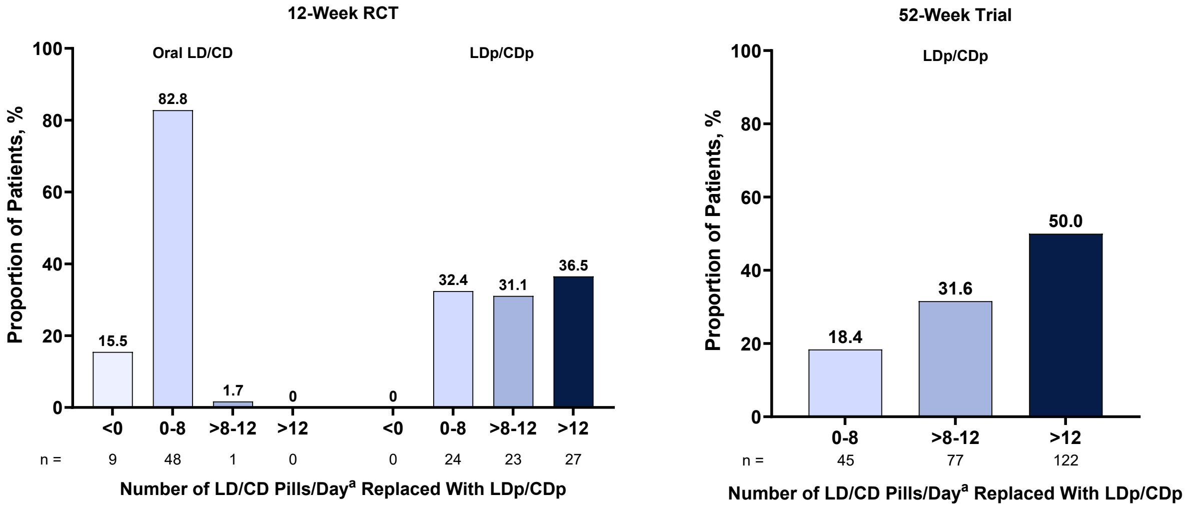

Supplementary Figure 2. *Reduction in concomitant PD medication classes in patients receiving combination therapy in the 96-week OLE study.*

BL, baseline; OLE, open-label extension; PD, Parkinson's disease.

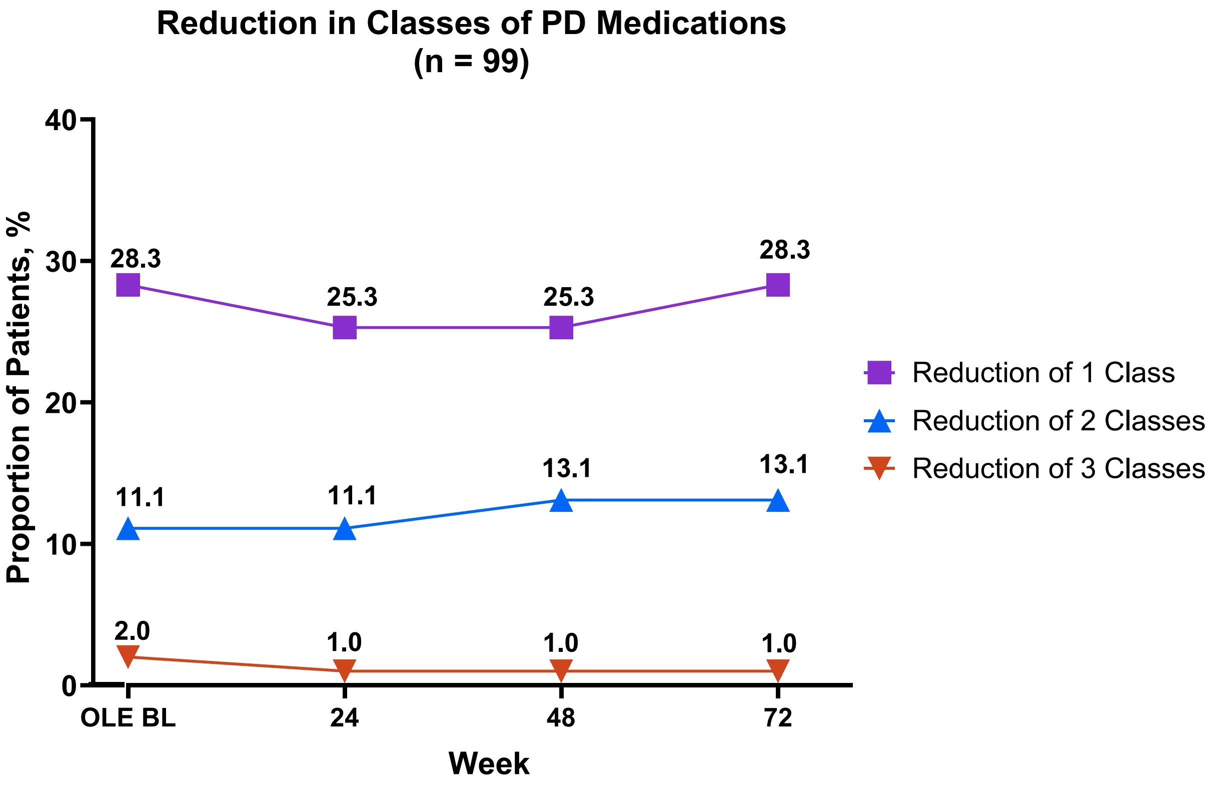

Supplementary Figure 3. *Mean daily levodopa equivalent dose in the (**A**) 52-week trial and (**B**) 96-week OLE.*

Combo, Combination therapy; Mono, monotherapy; OLE, open-label extension.

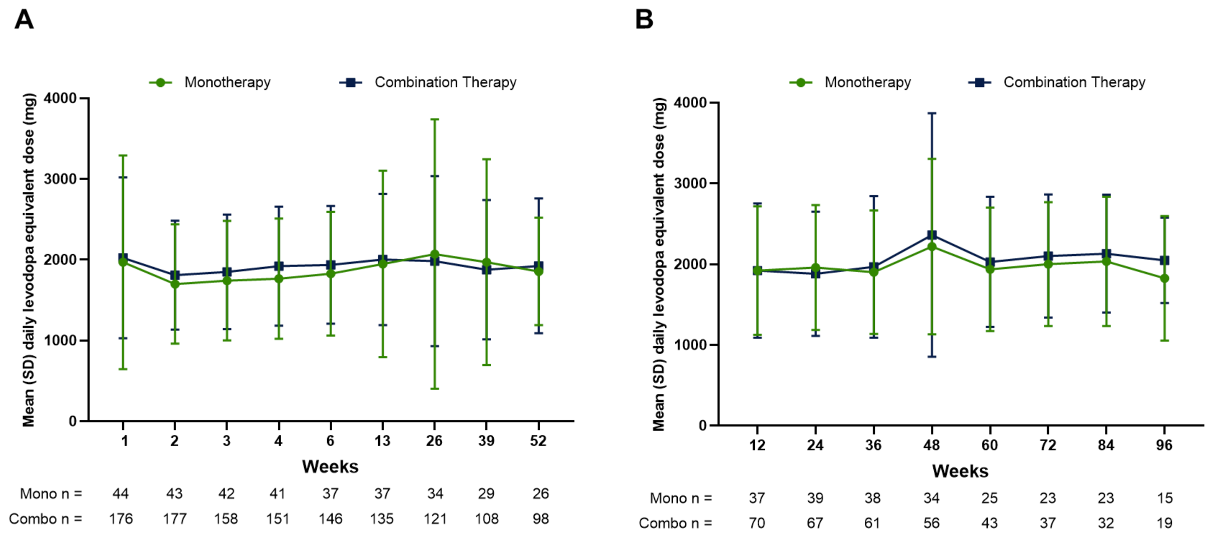

Supplement: Supplementary file 1 — Table S1. Documented use of oral rescue LD/CD (levodopa/carbidopa) at the end of the treatment period in the monotherapy groups. Table S2. Use of concomitant medications at baseline and end of the treatment period. Table S3. Summary of most common TEAEs (treatment‐emergent adverse events). Figure S1. Reduction in oral LD/CD pill burden. Figure S2. Reduction in concomitant PD medication classes in patients receiving combination therapy in the 96‐week OLE study. Figure S3. Mean daily levodopa equivalent dose in the (A) 52‐week trial and (B) 96‐week OLE. [file MDC3-13-181-s001.pdf]
